# Rate of change in solar insolation is a hidden variable that influences seasonal alterations in bipolar disorder

**DOI:** 10.1002/brb3.2198

**Published:** 2021-06-01

**Authors:** Sandra J. Rosenthal, Travis Josephs, Oleg Kovtun, Richard McCarty

**Affiliations:** ^1^ Department of Chemistry Vanderbilt University Nashville TN USA; ^2^ Department of Pharmacology Vanderbilt University Nashville TN USA; ^3^ Department of Chemical and Biomolecular Engineering Vanderbilt University Nashville TN USA; ^4^ Neuroscience Program Vanderbilt University Nashville TN USA; ^5^ Department of Psychology Vanderbilt University Nashville TN USA

## Abstract

The consensus in the literature is that bipolar disorder is seasonal. We argue that there is finer detail to seasonality and that changes in mood and energy in bipolar disorder are dictated by the rate of change of solar insolation.

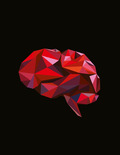

There is a general consensus in the literature that symptoms of bipolar disorder (BD) display a seasonal pattern of onset in some individuals (Fellinger et al., 2017; Geoffroy et al., 2014; Kim et al., 2015). A critical unresolved issue concerns how seasonality is expressed in BD. In the spring, increased energy levels are followed by sustained elevations in mood, which may lead to symptoms of hypomania or mania as the summer solstice approaches and the longest days of the year occur. In contrast, energy levels wane in the fall, followed later by a depressed mood, which can manifest as serious depression with the approach of the winter solstice when the shortest days of the year occur (McCarty, 2020; Rosenthal et al., 2020).

These predictable changes in daylength and ambient temperature result from the 23.5° tilt of the earth on its rotational axis relative to its annual elliptical orbit around the sun. As distance from the equator increases, daylight increases in summer and decreases in winter, eventually resulting in near‐continuous light and continuous darkness, respectively, near the poles. There are several ways to quantify the amount of sunlight in a specified area. For example, solar insolation is the amount of solar radiation, measured in Watts (Joules/second, or energy per second) incident per square meter at a given location (Hut et al., 2013). In addition, the U.S. National Aeronautics and Space Administration (NASA) provides data for global horizontal irradiance (GHI) through a publicly accessible web site. GHI combines total solar radiation incident on a horizontal surface and includes direct perpendicular irradiance, diffuse horizontal irradiance, and ground‐reflected radiation. Simply put, this is a measure of the sunlight one detects on a daily basis (Li et al., 2015). NASA data can be localized down to an area of approximately 11 square miles or 770 square kilometers. Solar insolation is subject to a number of variables, and even cities at the same latitude may differ in their annual patterns of solar insolation. For example, Halifax, Nova Scotia in Canada (44.65^o^N) and Rochester, Minnesota in the United States (44.01^o^N) have quite different spring time patterns of solar insolation. Similarly, Buenos Aires, Argentina (34.6°S) and Cape Town, South Africa (33.9^o^S) differed in the month in which maximum positive change in GHI occurred (November and January, respectively). In addition, solar insolation paradoxically increases in October in Los Angeles, California as the Santa Ana winds push the marine layer out into the Pacific Ocean. Finally, seasonal fires and volcanic eruptions also cause transient disturbances in solar insolation that are captured in the NASA dataset (Rosenthal et al., 2020).

One can, in general, track changes in solar insolation from day to day by quantifying the amount of sunlight gained or lost compared to the previous day. These values tend to be maximal at the spring and fall equinoxes in the northern hemisphere and are greatly reduced at the solstices. Thus, the change in solar insolation from day to day is not constant throughout the year. Consider this simple illustration: at both the winter and summer solstices, the earth is in the gentle bend of its elliptical orbit, and solar insolation does not change noticeably from one day to the next. Heading from a solstice to an equinox, there is a substantial uptick in the rate of change in solar insolation from month to month. We propose that *the rate of change in solar insolation* is a “hidden variable” that signals changes in energy levels and mood in BD. Our ultimate goal with this experimental approach is to provide a quantitative tool for improving the diagnosis, treatment, and self‐management of BD.

Several lines of evidence have documented disruptions in circadian and seasonal rhythms in patients with BD. Sleep disturbances occur in BD patients during depressive episodes and have included actigraphic measures of increased sleep latency, hypersomnia, and reduced sleep efficiency. During manic episodes, BD patients experience decreased need for sleep without daytime fatigue and higher energy levels. Sleep disturbances also occurred in BD patients when in remission, including longer sleep durations, longer latencies to fall asleep, and more frequent awakenings after sleep onset compared to healthy controls. In addition, BD patients in remission displayed greater variability in daily patterns of sleep and wakefulness compared to controls, with a preference for an evening chronotype, a more variable bedtime, shorter duration of sleep, and reduced self‐reported sleep satisfaction (Dallaspezia & Benedetti, 2021; Hensch et al., 2019).

BD patients also display evidence of disruptions in regulation of melatonin secretion from the pineal gland. Plasma levels of melatonin display a circadian rhythm, with higher levels in the nighttime and much lower levels during the day. In addition, plasma melatonin levels also reflect seasonal changes in photoperiod, with higher peak levels and more prolonged periods of secretion during the longer nights of winter versus the shorter nights of summer. Clinical studies have revealed that pineal melatonin secretion is hypersensitive to light, is maintained at lower levels, and peaks later at night in BD patients compared with healthy controls (Bradley et al., 2017; Maruani et al., 2018).

Another approach to study circadian changes in BD has involved studies of lymphocytes, fibroblasts, or epithelial cells obtained from BD patients and controls and maintained in culture. Several reports of altered patterns of regulation of circadian clock genes in BD patients have appeared, and clock genes may play a critical role in lithium's mood‐stabilizing effects in BD. Consistent with this view, lithium, a first‐line treatment for BD, unmasked differences between lithium responders and nonresponders in the regulation of circadian clock genes using cultured skin fibroblasts. Studies of circadian clock genes using cellular models may open up new avenues to explore underlying mechanisms relating to lithium's effects on circadian rhythms and its use as a mood stabilizer in patients with BD (McCarthy et al., 2016, 2019).

Taken together, the compelling evidence in support of circadian disturbances in BD may also feed into seasonal influences relating to the onset of symptoms of bipolar mania or bipolar depression. Studies of the association between hospital admissions of BD patients and solar radiation, maximum temperature, and seasonal patterns in photoperiod have shown that manic episodes tend to peak during spring and summer months, with a minor peak in autumn (Aguglia et al., 2017, 2018, 2019). In contrast, depressive episodes displayed peaks in early winter and less frequently in summer, with mixed episodes peaking in early spring or summer. Manic episodes and depressive episodes displayed strong seasonal patterns, with the latter being associated with a more complex clinical proﬁle of the bipolar II subtype, more relapses, and rapid cycling between mood states. In addition, BD patients displayed greater seasonal variations in mood, social interactions, weight gain, and sleep patterns.

Bauer and his colleagues have taken a creative approach to study the seasonal influences on expression of symptoms of mania and depression by collecting data on bipolar I patients from multiple sites in the northern and southern hemispheres, across a wide distribution of latitudes (Bauer et al., 2014, 2017). Their rationale was that individuals who live greater distances from the equator would experience much greater changes in levels of solar insolation from winter compared with summer months and this greater amplitude of seasonal changes in light would impact BD symptom expression. This international team of investigators obtained data from hospital records or direct interviews whenever possible, with a focus on the age of onset and polarity of the first episode of BD, the geographic location, and any family history of mood disorders. To standardize results across all locations, 6 months were subtracted from locations in the southern hemisphere. Their analyses revealed a dramatic inverse relationship between maximum monthly increase in solar insolation and age of onset of BD, and this relationship was reduced by about 50% but still significant in patients with no family history of mood disorders. Specifically, the age of onset of symptoms of BD occurred much earlier in individuals who resided in locations that were distant from the equator, where the greatest monthly changes in solar insolation occur (Rosenthal et al., 2020). The effect, although still highly significant, was one‐third smaller for initial episodes of mania compared to initial episodes of depression. The maximum monthly increase in solar insolation occurred in springtime for all sites where onset of BD was tracked, with 40% occurring between February and March, 38% between March and April, and 11% between April and May. A similar data collection strategy revealed a 5‐year average difference in age of onset of BD between the locations with the largest versus the smallest monthly increases in solar insolation. In addition, suicide attempts increased by 49% in going from locations closer to the equator with minimal changes in solar insolation from winter to summer months compared with locations at greater distances from the equator with maximal changes in solar insolation from winter to summer months (Bauer et al., 2014).

The results of these multisite studies provide clear evidence that the magnitude of seasonal changes in solar insolation impacts the age of onset of symptoms of BD as well as the risk of suicide attempts. But a key question still remains: what aspect of seasonal changes in solar insolation is measured as light is detected by specialized intrinsically photoreceptive retinal ganglion cells (ipRGCs) that express melanopsin and that project to neurons in the suprachiasmatic nucleus (SCN), the master clock of the body? The SCN then projects via a multisynaptic pathway, eventually influencing the synthesis and secretion of melatonin from the pineal gland. Is the SCN simply measuring the intensity of ambient light, or is something more subtle occurring? It is also possible that seasonal patterns of secretion of melatonin from the pineal gland reflect rates of change in solar insolation.

Recently, we argued that the SCN may play a critical role in measuring the *rate of change* in solar insolation over the course of a year rather than simply the month‐to‐month average amounts of solar insolation (Rosenthal et al., 2020). To generate evidence to address this hypothesis, we selected 51 cities for our study, including 10 cities from south of the equator. We utilized the NASA website to obtain monthly measures of global horizontal irradiance (GHI in W/m^2^) for each month of calendar year 2017 for each of these locations. We computed monthly values for rate of change in GHI for each site using a MATLAB script. Sites in the northern hemisphere tended to attain maximum positive rates of change in GHI in the spring (February–May) and maximum negative rates of change in GHI in the fall (September–November). In contrast, locations in the southern hemisphere tended to attain maximum positive rates of change in GHI in the fall (September–November) and maximum negative rates of change in GHI in the spring (March–May). We subtracted 6 months from the 10 sites in the southern hemisphere to permit combining data for all 51 sites. Most sites had maximum positive rates of increase in GHI between February and May and maximum negative rates of increase in GHI occurred between September and November. An intriguing question awaits further experimentation: to what extent are patterns in BD symptoms dominated by the rate of change in solar insolation, as opposed to simply the magnitude of the change in photoperiod from one month to the next?

Focusing on the rate of change in solar insolation may have important implications for clinicians who care for BD patients. An important variable for consideration by BD patients is the pattern of seasonal changes in solar insolation where they reside. Simply knowing one's latitude is not enough, as locations that are similar distances from the equator may have quite different patterns of solar insolation over the course of a year. Having a detailed knowledge of local rates of change in solar insolation over the course of a year would provide an additional element of self‐management and predictive planning for individuals with BD. An example of a useful resource to obtain such data is SolarAnywhere®, a software maintained by Clean Power Research that provides on‐demand access to bankable solar insolation data captured by geosynchronous orbiting satellites with spatial resolution as high as 1 km × 1 km (0.01 × 0.01 deg. gridded tile) at 10‐min, 5‐min, or 1‐min intervals. SolarAnywhere® is widely used for real‐time solar resource assessment and forecasting in the photovoltaic industry and could be readily repurposed for the clinical setting. Level of medication, psychotherapy, and/or chronotherapies could be personalized such that therapeutic strategies employed during the transition from winter to spring (positive rates of change in solar insolation) might differ from strategies employed during the transition from summer to fall (negative rates of change in solar insolation) (Rosenthal et al., 2020). This same line of reasoning might also apply to individuals with depressive disorder with seasonal pattern, formerly known as seasonal affective disorder (SAD) (Garbazza & Benedetti, 2018; Wirz‐Justice, 2018).

Although modern humans have had the ability to supplement their interior and exterior environments with artificial light at night for several centuries, many of the regulatory components of circadian clocks and the neural circuits and hormonal systems they influence have evolved over millions of years to detect daily and seasonal changes in photoperiod and have been conserved in modern humans. Daily activities of most humans still provide subtle cues regarding the rate of change in solar insolation across the seasons. We suggest that these cues relating to rate of change in solar insolation constitute a hidden variable that impacts the onset, progression, and annual timing of symptoms of BD and may influence the response to various therapeutic interventions. Further consideration might also include the intensity of bipolar symptoms dependent on both the rate of change of solar insolation and the geographic location.

## CONFLICTS OF INTEREST

The authors declare no conflicts of interest.

### PEER REVIEW

The peer review history for this article is available at https://publons.com/publon/10.1002/brb3.2198.
